# Supplementary material for: Structural proteomics defines a sequential priming mechanism for the progesterone receptor
Source: Res Sq. 2024 Nov 14:rs.3.rs-5199635. Preprint. [Version 1] doi: 10.21203/rs.3.rs-5199635/v1 (PMC11601812; doi:10.21203/rs.3.rs-5199635/v1)
Supplement: Supplement 1 [file NIHPPRS5199635V1-supplement-1.pdf]

Supplementary Files

This is a list of supplementary files associated with this preprint. Click to download.

- SequentialPrimingPRSupplement.docx
- ExtendedData.docx
